# Aortic Valve Replacement: A Case of Aortic Valve Insufficiency Secondary to Infective Endocarditis

**DOI:** 10.7759/cureus.46628

**Published:** 2023-10-07

**Authors:** Mudit Dutta, Rushi K Patel, Eric J Basile, Miguel A Belaunzaran

**Affiliations:** 1 Internal Medicine, University of Florida College of Medicine, Gainesville, USA; 2 Medical School, Dr. Kiran C. Patel College of Allopathic Medicine, Davie, USA

**Keywords:** infective endocarditis, aortic valve insufficiency, tavr (transcatheter aortic valve replacement), surgical aortic valve replacement (savr), endocarditis

## Abstract

Acute aortic valve insufficiency (AAVI) is a pathologic medical condition that has a presentation on a spectrum of severity and is attributable to numerous etiologies. Most often, it is caused by infective endocarditis, which depending on the patient’s clinical status may require treatment with either surgical aortic valve replacement (SAVR) or transcatheter aortic valve replacement (TAVR). This case presents a patient with acute aortic valve insufficiency secondary to infective endocarditis, requiring intervention. Further, it also provides real-time use of the general guidelines used in the determination of SAVR vs. TAVR candidacy. This case will further help providers in the cardiology realm to identify this presentation and increase comfort in referring to existing guidelines, as well as highlight where the current guidelines appear limited.

## Introduction

Acute aortic valve insufficiency (AAVI), often linked to infective endocarditis, poses a critical health risk that may potentiate urgent treatment. While some patients respond well to medical treatment, many require surgery to restore valve function and prevent complications. Aortic valve replacement (AVR) emerges as the preferred surgical solution for those with AAVI due to endocarditis, offering two main options: transcatheter aortic valve replacement (TAVR) and surgical aortic valve replacement (SAVR). The choice between these approaches depends on individual patient factors such as the severity of valve dysfunction, infection extent, comorbidities, and the patient's overall health. 

The decision of whether to proceed with surgery and, if so, when to perform AVR in AAVI cases remains a matter of ongoing debate [1-3]. In this paper, we present a case where acute endocarditis led to subacute aortic valve insufficiency, resulting in a bioprosthetic SAVR. This case highlights the complex decision-making process surrounding surgical interventions for AAVI secondary to endocarditis, offering insights from one specific case and the factors that influenced clinical decisions.

## Case presentation

**Figure 1:**
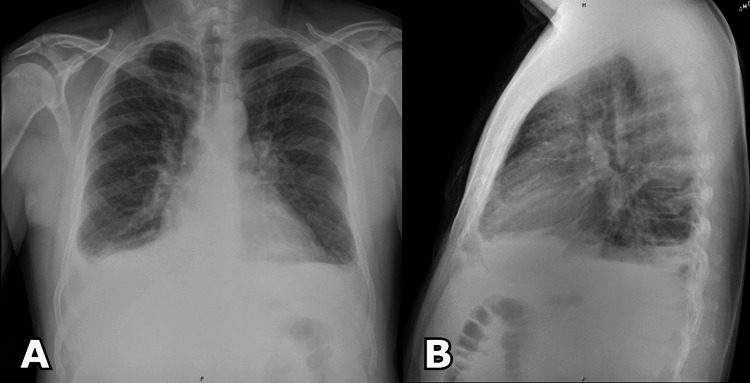
Anterior-posterior (A) and lateral (B) chest X-rays demonstrating pulmonary vascular congestion, cardiomegaly, and bilateral pleural effusions.

**Figure 2:**
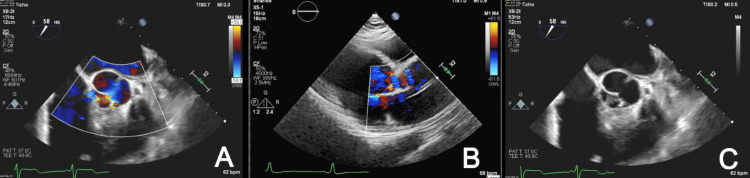
Transesophageal echocardiogram demonstrating aortic regurgitation during diastole in the parasternal short axis on the Doppler (A), aortic regurgitation in the parasternal long axis with the Doppler (B), and vegetation on the aortic valve (C).

A 48-year-old gentleman with a past medical history of obstructive sleep apnea and polysubstance use (including intravenous cocaine and methamphetamine) presented to the emergency department with progressive shortness of breath and fatigue. Associated symptoms included dyspnea on exertion, orthopnea, and abdominal and lower extremity swelling. Of note, the patient’s previous medical care was in Mexico, where he was diagnosed with congestive heart failure and treated with antibiotics for presumed valvular disease without a specified timeline. His home medications included metoprolol tartrate, spironolactone, and atorvastatin. The patient was hemodynamically stable with notable exam findings including diffuse lung crackles bilaterally, 5/6 diastolic heart murmur, and mild pitting edema of both lower extremities. Chest x-ray revealed cardiomegaly and pulmonary vascular congestion (Figure 1). He was ultimately admitted to the cardiology service for newly diagnosed decompensated heart failure and initially started on diuretics. Transthoracic echocardiogram (TTE) revealed severe aortic regurgitation, left ventricular dilation and hypertrophy, and normal right and left-sided ventricular function. Subsequent transesophageal echocardiogram (TEE) confirmed the aortic regurgitation with eccentric jet and cited the presence of large-sized vegetation (4.7 cm) with multiple filamentous projections on the ventricular aspect of the left coronary cusp leaflet along with holo-diastolic flow reversal in the ascending aorta (Figure 2). Cardiac catheterization revealed normal hemodynamics and no sign of coronary artery disease.

The cardiothoracic surgery service was consulted for evaluation and consideration of the patient's eligibility for aortic valve replacement given his entire clinical picture and history of polysubstance abuse (albeit over one year ago). Due to the patient’s preference to not be on lifelong warfarin and the associated valve sound from having a mechanical valve, the patient elected for the placement of a bioprosthetic valve. The patient was aware of the valve’s efficacy being limited to 10-15 years and that it may require redoing SAVR. On the ninth hospital day, surgical bioprosthetic aortic valve replacement was performed with initial annular enlargement of the aortic root that would permit the largest possible prosthesis to facilitate future valve management in the setting of a narrow sinotubular junction (STJ). Of note, intraoperative findings included infective destruction of the left and non-coronary leaflets with stringy vegetation and a lack of annular involvement or root abscess. After valve placement, supravalvular stenosis was noted intraoperatively and attributed to the patch aortotomy closure, which was resolved with subsequent vertical division of the STJ and the original patch was followed with augmentation that involved further aortic root reconstruction. Due to concern for bacterial seeding from the vegetation noted intraoperatively, the patient was placed on a course of vancomycin and cefepime for a total of six weeks. Ultimately, intraoperative bacterial, anaerobic, acid-fast bacillus, and fungal cultures did not grow any organisms. Pathologic examination of the explanted aortic valve leaflets found calcific degeneration and focal acute inflammation without any fungal forms identified on the silver stain. His follow-up TTE demonstrated normal ejection fraction and normally functioning aortic valve prosthesis.

## Discussion

The presented case emphasizes the importance of in-depth evaluation and serves as another data point to contribute to the body of literature surrounding the use of SAVR in acute aortic regurgitation secondary to infective endocarditis; the patient’s symptomatic progression, history of intravenous drug use with previous endocarditis, and mild left ventricular hypertrophy directed the treating team toward a diagnosis of recurrent endocarditis of the aortic valve. Although no organisms were identified on the work-up, this was likely attributable to numerous previous rounds of antibiotics. 

Due to his worsening heart failure symptoms and echocardiogram findings of severe aortic regurgitation, the cardiology service recommended valvular intervention and presented both TAVR and SAVR to the patient as potential options noting that bioprosthetic SAVR would be preferable in the absence of other significant comorbidities. He underwent the procedure without complications, and the follow-up examination showed marked improvement in both clinical and echocardiographic findings. 

In this patient subset with acute aortic insufficiency, it is imperative to immediately risk stratify the patient's condition to elucidate whether or not the condition warrants urgent intervention. The European Society of Cardiology (ESC) is one of the only prominent organizations that provide guidelines on the appropriateness of surgical intervention in the setting of endocarditis with aortic regurgitation. Namely, the patient is a candidate for early surgical intervention-defined as within 48 hours of diagnosis, if the patient meets any of the following criteria: Hemodynamic instability due to severe aortic regurgitation, severe left ventricular dysfunction with an ejection fraction less than 30%, large mobile vegetations greater than one centimeter in size, persistent bacteremia or fever despite appropriate antibiotic therapy, and evidence of perivalvular extension of infection in the form of abscess, fistula, or pseudoaneurysm [1-3]. However, these guidelines do not indicate a preference for specific timing, even within those 48 hours. This is even less clear for patients who only have one or are on the border of meeting one of the above criteria. 

The timing of AVR in patients with AAVI secondary to endocarditis is controversial, and there is no clear consensus on the optimal timing of surgery. The 2020 ACC/AHA guidelines for managing patients with valvular heart disease only broadly state that surgery should not be delayed, especially if there is hypotension, pulmonary edema, or evidence of low flow [3]. Additionally, previous 2016 American Association for Thoracic Surgery guidelines for surgical treatment of infective endocarditis state that once an indication for surgery is established, the operation should be performed as soon as possible; the timing of surgery may be further complicated in patients with significant comorbidities like stroke and neurologic deficits [4]. However, in a less symptomatic patient who is otherwise clinically stable, there is no clear consensus on whether or not they should have early surgical repair versus prolonged medical management before surgical repair.

The present literature is still deciphering which patient populations benefit more from TAVR versus SAVR. Generally, these cases are evaluated on a case-by-case basis with particular evaluative emphasis on high-risk surgical patients, those with limited surgical options, hemodynamic instability, and valvular anatomy. Though, in some reviews, there is anecdotal evidence and some expert opinion that points to SAVR as the preferred option for this patient subset as it allows for complete removal of the infected valve and surrounding tissue as well as easier access to other affected cardiac structures. There is additionally a seemingly lower risk of 30-day mortality, stroke, and aortic valve reoperation in the SAVR group compared to those undergoing TAVR [5,6]. It should be noted, however, that these cited studies are relatively low-powered and that this is not reflective of general expert consensus based on statistical evidence.

## Conclusions

The presented case describes a clinical scenario that would benefit from more specific guidelines regarding when to perform surgery as well as to determine whether TAVR or SAVR is more appropriate for this specific patient population. Though the patient was deemed a surgical candidate due to presumed endocarditis with severe acute aortic regurgitation with refractory pulmonary edema, there was no clear consensus guidance on whether TAVR or SAVR was preferred. This case is limited by only being one data point, but it highlights the need for future research to be performed in order to draw more robust conclusions for patients with acute aortic valve insufficiency secondary to infective endocarditis; specifically, more research should be performed to determine if earlier intervention yields better outcomes in this patient subset, as well as which type of intervention produces the best long-term outcomes. More generally, this research should serve to aid in refining current guidelines on when to perform SAVR or TAVR, or the superiority of one over the other.
